# Role of Feed Forward Neural Networks Coupled with Genetic Algorithm in Capitalizing of Intracellular Alpha-Galactosidase Production by *Acinetobacter* sp.

**DOI:** 10.1155/2014/361732

**Published:** 2014-08-31

**Authors:** Sirisha Edupuganti, Ravichandra Potumarthi, Thadikamala Sathish, Lakshmi Narasu Mangamoori

**Affiliations:** ^1^Centre for Biotechnology, Institute of Science and Technology, Jawaharlal Nehru Technological University Hyderabad, Andhra Pradesh 500 085, India; ^2^Department of Chemical Engineering, Monash University, Clayton, VIC 3800, Australia; ^3^Bioengineering and Environmental Centre, Indian Institute of Chemical Technology, Hyderabad, Andhra Pradesh 500 607, India

## Abstract

Alpha-galactosidase production in submerged fermentation by *Acinetobacter* sp. was optimized using feed forward neural networks and genetic algorithm (FFNN-GA). Six different parameters, pH, temperature, agitation speed, carbon source (raffinose), nitrogen source (tryptone), and K_2_HPO_4_, were chosen and used to construct 6-10-1 topology of feed forward neural network to study interactions between fermentation parameters and enzyme yield. The predicted values were further optimized by genetic algorithm (GA). The predictability of neural networks was further analysed by using mean squared error (MSE), root mean squared error (RMSE), mean absolute error (MAE), mean absolute percentage error (MAPE), and *R* ^2^-value for training and testing data. Using hybrid neural networks and genetic algorithm, alpha-galactosidase production was improved from 7.5 U/mL to 10.2 U/mL.

## 1. Introduction

Alpha-galactosidases (3.2.1.22) belong to the family of glycosyl hydrolases or glycosidases. These enzymes catalyze the hydrolysis of terminal alpha 1–6 linked galactose residues from simple and complex oligosaccharides and polysaccharides [1]. They are widely distributed in plants, animals, and microorganisms. Alpha-galactosidases find potential applications in food, pharmacological, and chemical industries. The enzyme has been used in food industry for enhancing the nutritional quality of legumes by degrading galactooligosaccharides that cause gas or flatulence [2]. It is also used to improve crystallization of sugar by removing raffinose from molasses in beet sugar industry [3] and in guar gum processing [4] and for enhancing bleaching of softwood along with mannanase in paper and pulp industry [5] and in processing of animal feed [6]. In humans, mutations in* gfA* gene lead to Fabry's disease, a rare X-linked recessive lysosomal storage disorder. Enzyme replacement therapy with *α*-galactosidase is considered a potential treatment for Fabry's patients [7]. In addition, the enzyme can also convert type “B” erythrocytes to type “O” erythrocytes [8] and is also used in xenotransplantation [9]. Microbial sources for alpha-galactosidase are being explored because of ease of cultivation and fermentation conditions. However, for cost-effective production, fermentation medium plays a vital role in the commercial production of enzymes. The nutritional requirements of each microorganism are varied and are regulated by physiological, biochemical, and genetic makeup of the organism [10]. Therefore optimization of fermentation medium is considered a crucial step for cost-effective production of the desired product. Traditional methods use one at a time method of approach that is laborious and time-consuming and it does not reflect interactions between different variables [11]. Experiments based on statistical methods are considered to be more economical and effective than traditional methods in understanding interaction between variables and minimizing the number of experiments. Response surface methodology (RSM) is one of the widely used statistical methods for the optimization of medium parameters [12–17]. RSM-based models can predict the relationship between a limited number of input and output parameters and hence cannot be applied for highly nonlinear processes [18]. Artificial neural networks (ANNs) and genetic algorithms (GAs) are termed as artificial intelligence that mimics the human with a given set of experimental criteria; GA enables identification of best alternative with goodness of fit by performing multiple random searches. ANN along with GA is widely used in various optimization studies, even in cases where the primary function under study is discontinuous, nondifferentiable, stochastic, or highly nonlinear [19, 20]. Currently, hybrid ANN-GA is being applied in optimizing various physical and nutritional fermentation parameters. This method has been applied to enhance the production of alkaline protease from* Bacillus circulans* and glutaminase from* Bacillus subtilis* [21, 22].

The present study focuses on enhancing the production of intracellular alpha-galactosidase from* Acinetobacter *sp. isolated from sugar cane waste by using hybrid artificial neural networks and genetic algorithm (ANN-GA). This is the first report of optimization of intracellular alpha-galactosidase using ANN-GA. A feed forward neural network (FFNN) together with back propagation was used for nonlinear modelling in this study to reduce the experimental error and subsequent optimization of enzyme production using genetic algorithm (GA).

## 2. Materials and Methods

### 2.1. Microorganisms

**Figure 1:**
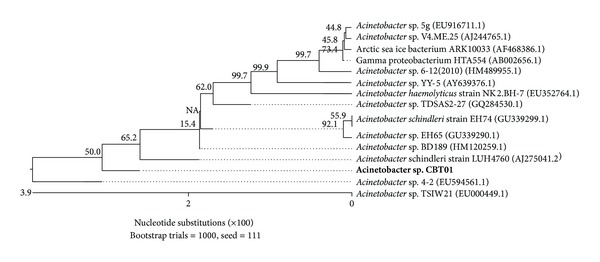
Phylogenetic analysis of* Acinetobacter *sp. CBT01 by ClustalW software (Accelrys, San Diego, CA, USA). The branching pattern was generated by the neighbor joining method.

Microorganisms were isolated from sugar cane waste. The isolate was observed to be gram-negative, short-rods, nonmotile, and nonspore forming bacteria. The isolate was positive for catalase and citrate but negative for nitrate reduction, H_2_S production, and oxidase and indole production. Based on morphological, biochemical, and 16s rRNA sequencing analysis, the isolate showing maximum intracellular alpha-galactosidase activity was identified as* Acinetobacter *sp. (Figure 1). The organism was grown at 36°C for 12 hours and maintained on agar slants at 4°C and was subcultured at 4-week interval.

### 2.2. Inoculum Preparation and Cell Lysis

A 24-hour-old culture 0.5% (w/v) inoculum was taken and inoculated into a 250 mL Erlenmeyer flask containing 100 mL sterile production media containing raffinose 25, tryptone 10, K_2_HPO_4_ 10, MgSO_4_·7H_2_O 1, and FeSO_4_·7H_2_O 1 in g l^−1^ (pH 7.0). The inoculated culture media were incubated at 36°C for 24 hours in shaking incubator at an agitation speed of 170 rpm. Cells were harvested from broth by centrifugation at 10,000 g and washed with 20 mM Tris buffer (pH 7.0). The cells were suspended in the same buffer containing 0.3% (w/v) lysozyme, 0.1% (w/v) Triton X 100, and 1 mM PMSF and incubated for 1 hour at 30°C. The cells were further disrupted by sonication. Cell debris was removed by centrifugation (10,000 g, 20 minutes, 4°C). Alpha-galactosidase activity was measured in the supernatant.

### 2.3. Alpha-Galactosidase Activity

Alpha-galactosidase activity was measured according to Dey and Pridham [1] in a reaction system containing 550 *μ*L of 20 mM Tris buffer (pH 7.2), 100 *μ*L of supernatant (enzyme preparation), and 250 *μ*L of 2 mM *ρ*-nitrophenyl-alpha-D-galactopyranoside (*ρ*NPGal). The reaction mixture was incubated at 50°C for 10 minutes and the reaction was stopped by addition of 1 mL of 0.2 mM Na_2_CO_3_. The absorbance was read at 405 nm. One enzyme unit (U) is defined as the amount of enzyme required to produce one *μ*mol of *ρ*-nitrophenol per minute under the above assay conditions.

### 2.4. Modelling and Optimization of Enzyme Production

#### 2.4.1. Data Sets

In the present study, the most promising factors which influence the alpha-galactosidase production were optimized by using the neural networks and genetic algorithms. Based on the preliminary studies (data not shown), temperature, pH, agitation speed, raffinose, tryptone, and K_2_HPO_4_ concentrations were found to be the most important parameters that influence alpha-galactosidase production from the isolated bacterial strain. The list of selected variables with their minimum and maximum concentrations was given in Table 1. A central composite design with 50 experiments was employed in the present study (Table 2). The data was divided into two sets comprising 40 observations used for training the network and 10 data sets used as testing data. The training data was used to compute the network parameters. The testing data was used to ensure robustness of the network parameters.

#### 2.4.2. Artificial Neural Networks

In the present study a multilayer perceptron (MLP) neural network was used. A feed forward neural network, which uses error backpropagation learning algorithm (BPNN), was constructed for modelling alpha-galactosidase production. The network consists of three layers of neurons, namely, an input layer, a hidden layer, and an output layer. All three layers are connected to the subsequent layers in the forward direction; the connections are termed as weights. The weights play a vital role in optimizing the data. Experimental conditions were chosen as inputs for the network whereas output is alpha-galactosidase activity. The number of the neurons in the hidden layer was optimized based on the trial and error method (examined from 3 to 18). All the data were normalized to −1 to +1. Scaled data are passed through the input layer and then data is propagated from input layer to hidden layer and finally to reach the targets (output layer) of the network. Every node in input and hidden layer is connected to the nodes in the subsequent layer. Each neuron in the hidden and output layer acts as a summing junction, which combines and modifies the inputs from the previous layer using the following equation:

(1) $$\begin{array}{c} Y_{i}=\overset{n}{\underset{j=1}{\sum }}x_{i}w_{ij}+b_{j}, \end{array}$$

where *Y*_*i*_ is net input to node *j* in hidden or output layer, *X*_*i*_ is outputs of previous layer, *W*_*ij*_ is weights between the *i*th node and *j*th node, *n* is number of neurons, and *b*_*j*_ is the bias associated with node *j*.

Sigmoid transfer function was used for the hidden layer and linear transfer function was used for the output layer to avoid error between observed and predicted values. During this process, Marquardt-Levenberg algorithm was used for training the network. Initially weight and bias values were taken randomly. However, in subsequent training steps, the weights and biases, in hidden and output layers, were adjusted in accordance with a convergence criterion to get the similarity in training and testing experimental values.

In order to evaluate the ANN output error, the coefficient of determination (*R* ^2^) was used, which describes the extent of variance in the modelled variables. The error was calculated based on difference between the experimental and predicted values. A popular measure such as mean squared error (MSE) or root mean squared error (RMSE), mean absolute error (MAE), and mean absolute percentage error (MAPE) was used to evaluate the ANN simulated data:

(2) $$\begin{array}{c} \text{MSE}=\frac{1}{n}\overset{n}{\underset{i=1}{\sum }}{\left(y_{p}-y_{e}\right)}^{2},\\ \text{RMSE}=\sqrt{\frac{1}{n}\overset{n}{\underset{i=1}{\sum }}{\left(y_{p}-y_{e}\right)}^{2}},\\ \text{MAE}=\frac{1}{n}\overset{n}{\underset{i=1}{\sum }}\left|y_{p}-y_{e}\right|,\\ \text{MAPE}=\frac{1}{n}\overset{n}{\underset{i=1}{\sum }}\frac{\left|y_{p}-y_{e}\right|}{y_{e}}, \end{array}$$

where *n* is number of experiments, *y*_*p*_ is ANN predicted value, and *y*_*e*_ is experimental value.

#### 2.4.3. GA Optimization

Genetic algorithm was used to search in different subspace and to locate the global maximum on the objective function surface. Optimization was performed with FFNN output values of weights and bias using fitness function:

(3) YOutput =WeightO    ×(21+e(−2×WeightH×Input  vector+Input  bias(bl))−1)  +Hidden  layer  bias(bH).

 Weight^*H*^ is weight on connections between input and hidden nodes.Weight^*O*^ is weight on connections between hidden and output nodes.

In this study, different parameters of GA optimization such as chromosome length (*L*_chr_) as 36, population size (*N*_pop_) as 36, crossover probability (*C*_*p*_) as 0.8, and mutation probability (*P*_mut_) as 0.01 were taken. Optimum conditions were selected after evaluation of GA for 500 generations (*Ng*_max⁡_ = 500) to achieve fine-tuned fermentation conditions in the given range of input parameters. Neural networks and genetic algorithm toolboxes of MATLAB 7.0 (The Mathworks, USA) were used in modeling studies.

## 3. Results and Discussion

**Figure 2:**
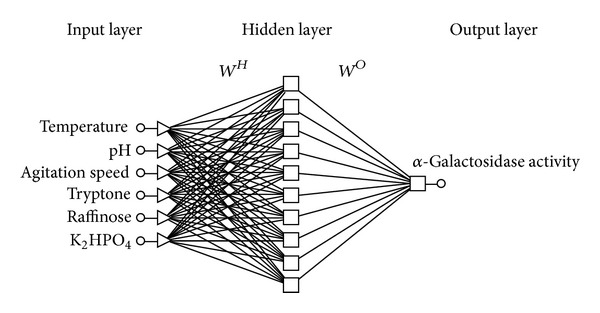
Feed forward neural network design used for optimization of alpha-galactosidase production. *W* ^*H*^ is weight of connection between input and hidden layer and *W* ^*O*^ is weight of connection between hidden and output layer.

In the present study, both physical and nutritional factors were chosen to optimize the enzyme production in shake flask. Table 2 depicts the experimental design along with experimental and predicted values of alpha-galactosidase production from* Acinetobacter *sp. From Table 2, it was observed that the enzyme production varied from the 3.3 to 7.5 U/mL under the various selected conditions. The observed minimum and maximum enzyme production indicate that the selected parameters have a greater influence on the alpha-galactosidase production. The data was further modelled with ANN and the conditions were optimized using the GA. The network was constructed by using the selected parameters and alpha-galactosidase production as input and output neurons. The selected six variables such as incubation temperature, pH, agitation speed, raffinose, tryptone, and K_2_HPO_4_ concentrations were chosen as input neurons in the input layer. Similarly the alpha-galactosidase production was set as output neuron in the output layer. The number of neurons in the hidden layer plays a vital role in the training time and generalization property of neural networks. Lesser number of neurons in the hidden layer would increase the training time whereas higher number of neurons in the hidden layer would cause overtraining and saturation of the network, which leads to false results. The number of neurons in a hidden layer depends on the complexity of the system being modelled. According to Sathish and Prakasham [22] the best approach to finding the optimal number of neurons in hidden layer is by trial and error method. In this study, the number of neurons in the hidden layer was varied from 3 to 18 and the optimal number chosen by the crossvalidation criterion with the number of epochs fixed at 1000 for all the structures studied. The neural network with 10 hidden neurons was found to have highest correlation and lowest MAPE and RMSE values.  Figure 2 depicts the constructed neural network topology “6-10-1” neurons in input, hidden, and output layers.

**Figure 3:**
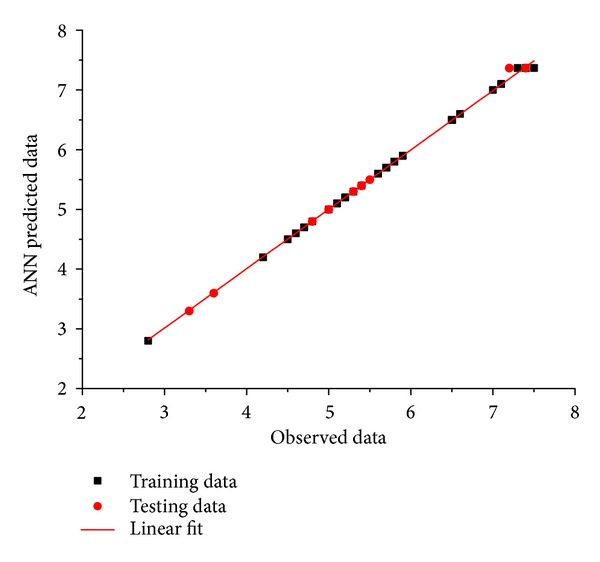
Correlation chart for experimental and predicted values of alpha-galactosidase activity.

The accuracy of the neural network based prediction can be calculated using the *R* ^2^ value based on the measured and predicted outputs in the training and test data. The calculated *R* ^2^ value was found to be 0.9994 indicating the model accuracy of the constructed ANN (Figure 3).  Figure 2 depicts good correlation between the experimental values and ANN predicted values, suggesting the accuracy of the ANN predictability of the nonlinear systems.

Further, the predictability of the neural networks was analyzed based on the MSE, RMSE, MAE, and MAPE of the training and testing data. The overall MSE (6.1 × 10^−4^), RMSE (2.47 × 10^−2^), MAE (3.4 × 10^−3^), and MAPE (4.4 × 10^−4^) of the training data suggested that the constructed network is suitable for the alpha-galactosidase production. This was further confirmed by testing data. The resultant data indicates a value of 2.8 × 10^−4^, 1.673 × 10^−2^, 1.4 × 10^−3^, and 1.8 × 10^−3^ for MSE, RMSE, MAE, and MAPE, respectively.

### 3.1. Interaction Influence of Selected Variables on the Alpha-Galactosidase Production

**Figure 4:**
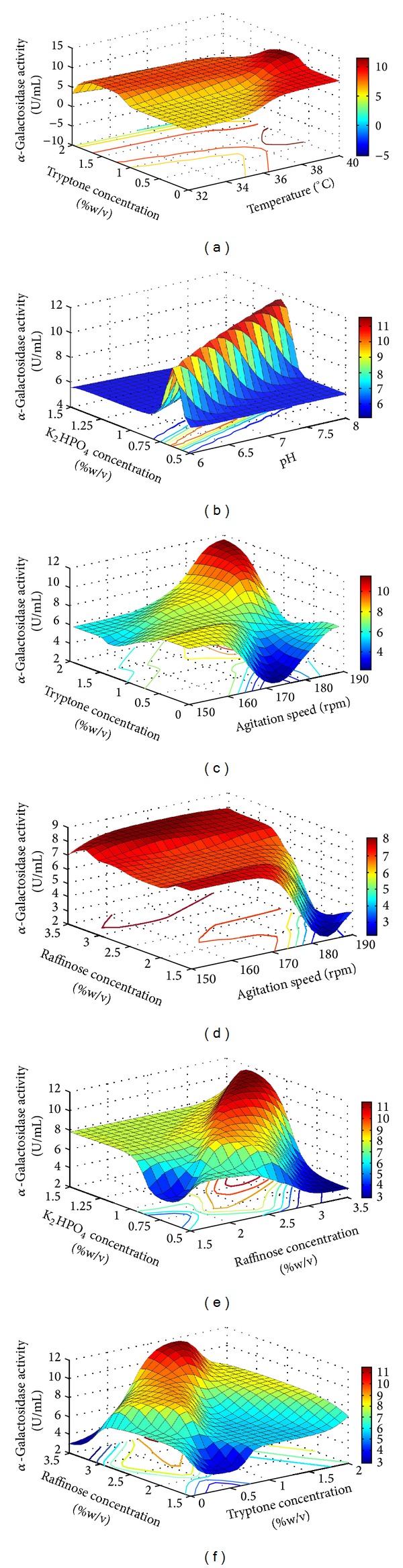
Effect of selected parameters interactions on alpha-galactosidase activity. (a) Tryptone versus temperature; (b) K_2_HPO_4_ versus pH; (c) tryptone versus agitation; (d) raffinose versus agitation; (e) K_2_HPO_4_ versus raffinose; (f) raffinose versus tryptone.

Analysis of interactions between different selective process parameters provides information on the concentration mediated regulatory role of alpha-galactosidase production.  Figure 4 shows the interactive influence of selected variables on alpha-galactosidase production.  Figure 4(a) depicts the influence of temperature with tryptone concentration indicating that alpha-galactosidase production increases with temperature up to 37°C. Similarly, studies on enzyme production at different pH values indicated that the production is better at neutral pH or pH slightly above neutral pH (Figure 4(b)). Similar findings have been reported in the literature (temperature 34 to 38°C and pH 6.8 to 7.5) for mesophilic bacteria. Mixing of the components in the media has a significant role in the microbial enzyme synthesis and secretion into external environment [22]. Figures 4(c) and 4(d) show the interaction influence of the agitation speed with tryptone and K_2_HPO_4_ concentration. From these graphs it can be concluded that, to achieve higher yields of the galactosidase, higher concentration of nutrients and higher agitation speed are needed. Figures 4(a), 4(c), and 4(f) depict the interaction influence of the tryptone with other process parameters as well as other nutrients. From these surface plots, it was observed that tryptone at 1–1.5% is favourable for the alpha-galactosidase production. The interaction influence of the carbon source (raffinose) and nitrogen source (tryptone) is depicted in Figure 4(f). It was observed that tryptone at 1% is suitable for the enzyme production. Figures 4(b) and 4(e) show the interaction influence of K_2_HPO_4_ with pH and raffinose.

### 3.2. GA Optimization and Validation Studies

The ANN output data was further optimized using GA. In order to obtain the best suitable conditions for alpha-galactosidase production, an objective function with weights and bias was used. Among 500 conditions generated by GA, four best suitable conditions were chosen and the validation experiments performed under these conditions (Table 3). From Table 3, it could be seen that the maximum intracellular alpha-galactosidase production was 10.2 U/mL which is 36% more than the maximum enzyme production in Table 2.

Alpha-galactosidase production titres vary for different microorganisms and are also influenced by microbial strain, enzyme localization, and physical and nutritional factors of fermentation medium. In the present study, the enzyme yield was increased from 7.5 (Table 2) to 10.2 U/mL (Table 3). Similar increase in enzyme production was reported in the case of* Streptomyces griseoloalbus* when optimized using RSM [23]. List of statistical methods and activity yield using various microorganisms is presented in Table 4. Similar trend was reported by several researchers working with alkaline protease [21], L-glutaminase production [22], and rifamycin production [24].

## 4. Conclusion

Alpha-galactosidase production by* Acinetobacter* sp. was optimized by using feed forward ANN-GA approach, selecting six different medium parameters. A cogent correlation of 0.9994 was obtained for observed and predicted values. Interactions of raffinose and temperature with other variables are considered to be significant for maximum enzyme production. The hybrid FFNN-GA approach showed excellent predictable accuracy and can also be used for other bioprocess methods.

## Supplementary Material

The supplementary data gives information about morphological, biochemical characteristics of the isolate identified as *Acinetobacter* sp. CBT01 isolated from the soil collected at sugar cane processing units. It is a fast growing organism and enters stationary phase at 5th of growth.

## Figures and Tables

**Table 1:** Selected factors and their minimum and maximum range chosen for intracellular alpha-galactosidase production.

Parameter	Low	High
Temperature (°C)	32	40
pH	6	8
Agitation speed (rpm)	150	190
Tryptone (g/100 ml)	0	2
Raffinose (g/100 ml)	1.5	3.5

**Table 2:** Experimental design and alpha-galactosidase activity (experimental and predicted) and error values.

Serial number	Temperature	pH	Agitation	Tryptone	Raffinose	K_2_HPO_4_	Alpha-galactosidase activity		
	(°C)		(rpm)	(g/100 mL)	(g/100 mL)	(g/100 mL)	Observed	Predicted	Error
1	34	6.5	160	0.5	2	0.75	2.80	2.5	0.28
2	34	6.5	160	0.5	3	1.25	4.70	4.78	−0.08
3	34	6.5	160	1.5	2	1.25	5.10	5.00	0.09
** ∗4**	**34**	**6.5**	**160**	**1.5**	**3**	**0.75**	**5.40**	**5.26**	**0.13**
5	34	6.5	180	0.5	2	1.25	5.70	5.79	−0.09
6	34	6.5	180	0.5	3	0.75	5.80	5.75	0.04
7	34	6.5	180	1.5	2	0.75	5.90	6.12	−0.22
8	34	6.5	180	1.5	3	1.25	6.60	6.69	−0.09
9	34	7.5	160	0.5	2	1.25	4.80	4.78	0.01
10	34	7.5	160	0.5	3	0.75	4.60	4.54	0.05
11	34	7.5	160	1.5	2	0.75	5.20	4.96	0.23
12	34	7.5	160	1.5	3	1.25	5.80	5.88	−0.08
** ∗13**	**34**	**7.5**	**180**	**0.5**	**2**	**0.75**	**3.60**	**3.45**	**0.14**
14	34	7.5	180	0.5	3	1.25	5.70	5.92	−0.22
15	34	7.5	180	1.5	2	1.25	6.50	6.54	−0.04
** ∗16**	**34**	**7.5**	**180**	**1.5**	**3**	**0.75**	**5.30**	**5.30**	**0**
17	38	6.5	160	0.5	2	1.25	5.30	5.26	0.03
18	38	6.5	160	0.5	3	0.75	6.50	6.42	0.07
19	38	6.5	160	1.5	2	0.75	5.80	5.54	0.25
20	38	6.5	160	1.5	3	1.25	4.70	4.81	−0.11
21	38	6.5	180	0.5	2	0.75	5.40	5.28	0.11
22	38	6.5	180	0.5	3	1.25	5.90	6.10	−0.20
** ∗23**	**38**	**6.5**	**180**	**1.5**	**2**	**1.25**	**5.40**	**5.42**	**−0.02**
24	38	6.5	180	1.5	3	0.75	5.60	5.58	0.01
25	38	7.5	160	0.5	2	0.75	5.20	5.07	0.12
26	38	7.5	160	0.5	3	1.25	6.50	6.24	0.25
27	38	7.5	160	1.5	2	1.25	5.60	5.61	−0.01
28	38	7.5	160	1.5	3	0.75	5.70	5.57	0.12
** ∗29**	**38**	**7.5**	**180**	**0.5**	**2**	**1.25**	**4.80**	**4.90**	**−0.10**
30	38	7.5	180	0.5	3	0.75	4.50	4.56	−0.06
31	38	7.5	180	1.5	2	0.75	4.20	4.08	0.11
** ∗32**	**38**	**7.5**	**180**	**1.5**	**3**	**1.25**	**3.30**	**3.55**	**−0.25**
33	32	7	170	1	2.5	1	5.10	5.11	−0.01
34	40	7	170	1	2.5	1	5.10	5.20	−0.10
35	36	6	170	1	2.5	1	5.70	5.74	−0.04
∗36	36	8	170	1	2.5	1	5.00	5.07	−0.07
37	36	7	150	1	2.5	1	5.60	6.23	−0.63
38	36	7	190	1	2.5	1	7.10	6.58	0.51
39	36	7	170	0	2.5	1	5.80	5.92	−0.12
40	36	7	170	2	2.5	1	6.50	6.49	0
** ∗41**	**36**	**7**	**170**	**1**	**1.5**	**1**	**5.50**	**5.89**	**−0.39**
42	36	7	170	1	3.5	1	7.00	6.72	0.27
43	36	7	170	1	2.5	0.5	5.00	5.65	−0.65
44	36	7	170	1	2.5	1.5	7.10	6.56	0.53
45	36	7	170	1	2.5	1	7.50	7.32	0.17
** ∗46**	**36**	**7**	**170**	**1**	**2.5**	**1**	**7.20**	**7.32**	**−0.12**
47	36	7	170	1	2.5	1	7.40	7.32	0.07
48	36	7	170	1	2.5	1	7.30	7.32	−0.02
** ∗49**	**36**	**7**	**170**	**1**	**2.5**	**1**	**7.40**	**7.32**	**0.07**
50	36	7	170	1	2.5	1	7.40	7.32	0.07

*Data used for testing.

**Table 3:** Best possible fermentation conditions and predicted and observed yields of enzyme alpha-galactosidase.

Serial number	Temperature	pH	Agitation	Tryptone	Raffinose	K_2_HPO_4_	Alpha-galactosidase activity	
	(°C)		(rpm)	(g/100 mL)	(g/100 mL)	(g/100 mL)	Predicted	Observed
1	35.1	6.8	180	1.2	2.5	1.1	9.5	9.6
2	36	7	180	1.4	2.8	1.3	10	9.9
3	37	7.2	183	1.1	2.4	1.7	10.5	10.2
4	36.5	7	175	1.4	2.5	1.3	9.4	9.8

**Table 4:** List of statistical methods used to enhance alpha-galactosidase production in various microorganisms.

Serial number	Organism name	I/E	Type of fermentation	Design	Design variables	Activity U/mL	Reference
1	*Streptomyces griseoloalbus *	E	Submerged	RSM (Box-Behnken Design)	pH, temperature, inoculum size, inoculum age, incubation period, agitation speed, carbon source, yeast extract, MgSO_4_ *·*7H_2_O, FeSO_4_, and salinity	50 U/mL	[23]

2	*Streptomyces griseoloalbus *	E	Solid-state	RSM	Inoculum size, moisture, and galactose	117 U/g of Fermented dry substrate of soyabean flour	[25]

3	*Aspergillus foetidus* ZU-G1	E	Solid-state	RSM	Wheat bran, soybean meal, KH_2_PO_4_, MnSO_4_ *·*H_2_O, and CuSO_4_ *·*5H_2_O	2207.19 U g(−1) dry matter	[26]

4	*Aspergillus foetidus* ZU-G1	E	Submerged	RSM	Soybean meal, wheat bran, KH_2_PO_4_, FeSO_4_ *·*7H_2_O, and the medium initial pH	64.75 U/mL	[27]
