# Supplementary material for: Role of Feed Forward Neural Networks Coupled with Genetic Algorithm in Capitalizing of Intracellular Alpha-Galactosidase Production by Acinetobacter sp
Source: Biomed Res Int. 2014 Aug 31;2014:361732. doi: 10.1155/2014/361732 (PMC4164808; doi:10.1155/2014/361732)

Supporting document for identification of Acinetobacter sp CBT01

Morphological and Biochemical Characterization:

The colony morphology of the isolate was observed to be circular with entire margins and opaque. The isolate is a gram-negative bacteria with short rods (cocobacilli) of 1-2μm in size (figure 1). The morphological and biochemical characteristics of isolate are given in the Table 1 and 2

Table 1: Morphological and physiological characteristics of isolate

Tests	Results
**Colony morphology**	
Configuration	Circular
Margin	Entire
Elevation	Convex
Surface	Smooth
Pigment	Off-white
Opacity	Opaque
Gram's reaction	negative
Cell shape	Rods
Size (μm)	1-2μm
Spore(s)	-
Motility	Non-motile
**Physiological characteristics**	
**Growth at temperatures**	
4°C	-
10°C	+
25°C	+

30°C	+
37°C	+
42°C	+
55°C	+
**Growth at pH**	
pH 4.0	-
pH 5.0	+
pH 6.0	+
pH 7.0	+
pH 8.0	+
pH 9.0	+
pH 10.0	+
pH 11.0	+

Table 2: Biochemical Characteristics of isolate

**Substrate, Test**	**Result**
Gram Staining	Negative
Citrate	Positive
Catalase	Positive
Gelatin liquefaction	Negative
Nitrate reduction	Negative
Oxidase	Negative
Acid production from	
Glucose	Positive
Sucrose	Positive
Mannose	Positive
Lactose	Negative
Indole	Negative
Methyl red	Positive
Voges-Proskauer	Negative

Figure 1 Scanning electron microscope of isolate at magnification of 10x.

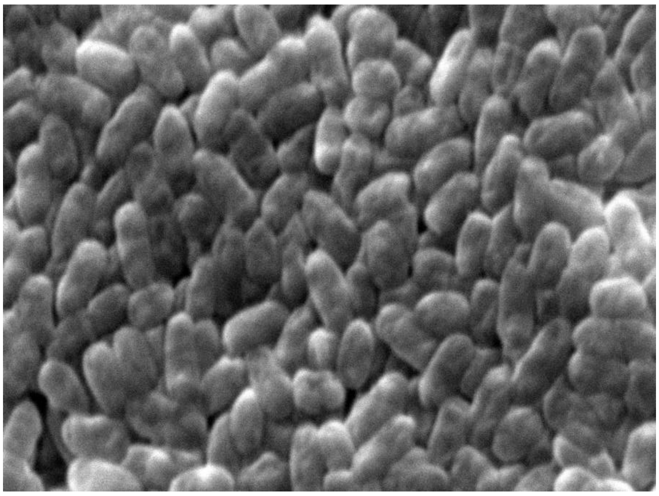

Supplement: Supplementary file 1 — The supplementary data gives information about morphological, biochemical characteristics of the isolate identified as Acinetobacter sp. CBT01 isolated from the soil collected at sugar cane processing units. It is a fast growing organism and enters stationary phase at 5th of growth. [file 361732.f1.pdf]
